# 

**Published:** 1881-07

**Authors:** 


### THE BISTOURY:

Thad S. Up de Graff, M. D., Editor & Proprietor.

### *CONTENTS FOR JULY, 1881.*

**CONTRIBUTIONS.** PAGE.

Suggestions Educational.......................BEN. H. PRATT,........................ 27

Our Cantata......................................AUSBURN TOWNER........................ 29

A Neglected part of Education.................J. J. KEYES.............................. 34

The War Cure.....................................J. B. CHANDLER .......................... 36

**MEDICAL ITEMS AND NEWS.**

Typhoid State—Asthma—Styptic Colloid—Pitting of Small Pox—Uterine Sedative—Iodoform—Diphtheria—Gelatine Bougies—Freckles—Cystitis—Diabetes—Antiseptic Catgut—Albuminous Obstruction of the Bladder—Toothache—Carbonated Laxative Water—Burns from Sulphuric Acid—Purgative for Consumptives—Gastric Flatulence, Acidity and Pyrosis—Facial Erysipelas—Subacute Gout or Rheumatism—Ringworm—Urticaria Produced by the Use of Tincture Pimpinallæ—Prolapsus Ani—Tic Douloureux—To Keep Leeches—Sodium Phosphate in Habitual Colic—Tape Worm—Acute Rheumatism—Perforation of Membrana Tympani from Ascaris Lumbricoides—Backache, Its Cause—Pilocarpine—Vaccine Virus.........................37 43

**OUR CORRESPONDENCE** Four Letters with answers.......................................... 18

**HOUSEHOLD HINTS AND RECIPES.**

To Silver Non-Metallic Substances—To Darken Oak Framing—Ashes as a Substitute for Emery—To Transfer Figures, Names, Pictures, etc., upon Glass—To Anneal Lamp Chimneys—Cement for Rubber—Liquid Glue—Salicylic Acid for Bee-Stings—Drinks for Fever Patients—Glucose—Worcestershire Sauce...............45 46

**EDITORIAL DEPARTMENT.**

Our Delay—H—Large Land Holder—Guiteau's Picture—Ancestral—Penalties—Effective Advertising—Why Defend—Probing for Bullets—Big Bass—Sea 'Side [Resorts—C. M. Beecher—Dr. Baxter and Dr. Bliss—The National Calamity—How to Rest.......................................47 52

